# Combining Electro-Osmotic Flow and FTA^®^ Paper for DNA Analysis on Microfluidic Devices

**DOI:** 10.3390/mi7070119

**Published:** 2016-07-14

**Authors:** Ryan Wimbles, Louise M. Melling, Kirsty J. Shaw

**Affiliations:** Faculty of Science and Engineering, Manchester Metropolitan University, Chester Street, Manchester M1 5GD, UK; ryan.wimbles@stu.mmu.ac.uk (R.W.); l.melling@mmu.ac.uk (L.M.M.)

**Keywords:** microfluidic, DNA purification, DNA amplification, FTA^®^ paper, electro-osmotic flow

## Abstract

FTA^®^ paper can be used to protect a variety of biological samples prior to analysis, facilitating ease-of-transport to laboratories or long-term archive storage. The use of FTA^®^ paper as a solid phase eradicates the need to elute the nucleic acids from the matrix prior to DNA amplification, enabling both DNA purification and polymerase chain reaction (PCR)-based DNA amplification to be performed in a single chamber on the microfluidic device. A disc of FTA^®^ paper, containing a biological sample, was placed within the microfluidic device on top of wax-encapsulated DNA amplification reagents. The disc containing the biological sample was then cleaned up using Tris-EDTA (TE) buffer, which was passed over the disc, via electro-osmotic flow, in order to remove any potential inhibitors of downstream processes. DNA amplification was successfully performed (from buccal cells, whole blood and semen) using a Peltier thermal cycling system, whereupon the stored PCR reagents were released during the initial denaturing step due to the wax barrier melting between the FTA^®^ disc and PCR reagents. Such a system offers advantages in terms of a simple sample introduction interface and the ability to process archived samples in an integrated microfluidic environment with minimal risk of contamination.

## 1. Introduction

The integration of nucleic acid purification and amplification techniques is often at the core of genetic analysis in microfluidic systems. The development of partially- and fully-integrated microfluidic systems for genetic analysis is detailed in excellent reviews by Park et al. [1] and Njoroge et al. [2]. One of the more challenging aspects of integration, which has still not been fully addressed, is the development of robust sample introduction methods to enable the easy introduction of biological samples, i.e., the real-world interface.

Biological specimens show great variability with regards to composition and informative value. For example, clinical samples, such as urine, which have a large volume, but low target analyte concentration, may be able to provide limited information. On the other hand, forensic blood specimens where the amount of sample available can be extremely limited (and any nucleic acids present may be degraded) can provide information on blood typing and DNA profiling, as well as overall blood pattern analysis. It is therefore important that any real-world interface is able to accommodate a wide range of samples with maximum efficiency. Pre-concentration of nucleic acids from large volume samples is commonly achieved through the use of solid-phase extraction matrices [3]. Alternative methods for large volume sample processing, up to 2 mL, to overcome the macro-micro interface include centrifugal devices for processing whole blood samples in order to separate out the plasma fraction [4] and digital microfluidic systems that use magnetic beads within small droplets to capture nucleic acids [5].

Purification methods used to prepare nucleic acids from biological matrices must be compatible with downstream processes, such as a polymerase chain reaction (PCR), if they are to be incorporated into truly integrative microfluidic systems. Challenges can include the confinement of any solid-phase matrix used, the carryover of reagents and the accuracy of timings for reagent movement [6]. In order to overcome these challenges, there has been a move away from more traditional silica-based solid-phase extraction protocols to a range of innovative solutions. Magnetic beads, for example, can be used to capture nucleic acids or specific target cell types, which, once captured, can be manipulated around the microfluidic device, through different reagents, by an external magnet [7,8]. Alternatively, the magnetic beads can be retained within a single chamber and the different reagent solutions flowed over the reversibly immobilised solid phase [9]. Kim et al. [10] demonstrated the use of a nanoporous aluminium oxide membrane for nucleic acid extraction, which makes use of more PCR-compatible chemicals allowing DNA extraction and amplification to be performed in a single chamber. Alternatively, nucleic acids eluted from a solid phase can be mixed with a flow of concentrated PCR reagent solution, but this mixing of two flow streams requires a well-characterised system in order to obtain the optimum elution fraction [11]. Electro-osmotic pumping has also been used to facilitate the integration of DNA extraction and amplification in a gel-based environment [12].

FTA^®^ paper (Whatman, Maidstone, UK) is a medium onto which a wide range of biological samples can be stored. It contains chemicals for cell lysis and protein denaturation and also protects any nucleic acids from oxidative and UV damage, as the paper contains a free radical scavenger [13]. Thus, it is an ideal medium for long-term storage and/or transport of biological samples. The use of FTA^®^ paper allows nucleic acids to be captured on a supporting membrane from which they can be directly amplified without the need for elution [14]. A limited number of studies have demonstrated the use of FTA^®^ paper within microfluidic devices. Such a methodology has successfully been applied to the detection of HIV-1 from oral fluids using the FTA^®^ paper in filtration mode to accommodate relatively large sample volumes prior to reverse transcription loop-mediated isothermal amplification [15]. Gan and colleagues demonstrated that untreated filter paper could be used as an extraction matrix within a microfluidic system. The filter paper was used to capture a range of biological samples, including whole blood left to dry onto FTA^®^ paper, whereupon cell lysis was achieved using 10 mM sodium hydroxide followed by neutralisation with 1 mM hydrochloric acid, then water. The filter paper, rather than the FTA^®^ paper itself, was then subject to direct PCR amplification both on- and off-chip [16]. A sample in-answer out system for integrated genetic analysis has also been proposed, which is able to take a 100-mm^2^ piece of FTA^®^ paper loaded with sample in a 1-mL syringe and transfer the biological sample directly onto the microfluidic device where complete analysis is performed [17].

The ability to store reagents within microfluidic devices offers numerous advantages, including a reduction in the complexity of the operational process, more compatibility with portable systems and a decreased risk of contamination. Reagent storage, particularly for molecular biology applications, has been demonstrated in liquid form, for example through the incorporation of stick [18] or blister packs [19], through encapsulation within gel matrices [12] or water-soluble nanofibers [20] and as freeze-dried reagents [21].

The work presented here exploits the direct use of FTA^®^ paper as both a means of simple sample introduction and as a conduit for the analysis of archived biological samples. The FTA^®^ discs provide a matrix for DNA purification in the microfluidic device, with the washing solutions transported using electro-osmotic flow (EOF). This was combined with on-chip storage of PCR reagents, by wax encapsulation, for integrated processing of archived biological samples by DNA amplification.

## 2. Materials and Methods

### 2.1. Manufacture and Functionalisation of Microfluidic Devices

**Figure 1:**
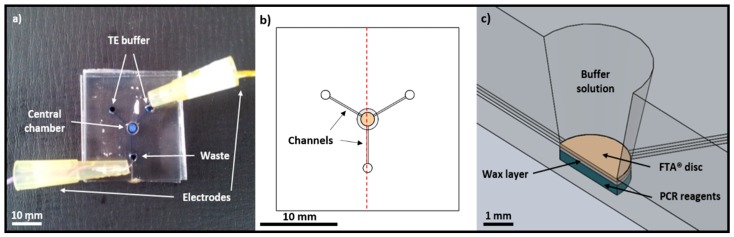
(**a**) Photograph showing the design of the microfluidic device used to perform integrated DNA purification and amplification experiments. The buffer wells are connected to the central chamber via 250-µm channels; (**b**) Schematic top-view showing the location of the channels, central chamber and reservoirs at the end of each channel. The dashed red line also indicates the cross-section view though which (**c**) occurs; (**c**) Schematic cross-section showing how the FTA^®^ paper discs are placed in the central chamber on top of a layer of wax-encapsulated PCR reagents.

Glass microfluidic devices were produced using standard photolithography and wet-etching techniques to produce the design shown in Figure 1a [22]. The channels in the 1-mm base plate were etched to a depth of 100 µm and the central chamber drilled to a total depth of 600 µm. The base plate was then thermally bonded to a 3-mm top plate. Access ports (1.5 mm diameter) were drilled in the top plate to accommodate reagents and electrodes for EOF. The central chamber for DNA extraction and amplification has a tapered hole (from 3 mm down to 2 mm) in the top plate to allow insertion of the FTA^®^ paper disc using a Micro-Punch (Harris, Maidstone, UK).

Silanisation of the PCR chamber was performed in order to prevent DNA polymerase adsorption, which would otherwise lead to PCR inhibition [23]. A 150 mM solution of trichloro-(1*H*,1*H*,2*H*,2*H*-perfluorooctyl)silane (Sigma-Aldrich, Dorset, UK) in 2,2,4-trimethylpentane (Fisher Scientific, Loughborough, UK) was incubated in the PCR chamber for 10 min. Solutions of 2,2,4-trimethylpentane, acetone and distilled water were then sequentially used to wash the device [24]. The microfluidic device was then stored in a desiccated environment until required.

### 2.2. Sample Preparation

Buccal samples were collected using Omni Swabs (Whatman), which were scrapped along the inside cheek of healthy volunteers. Each sheet of FTA^®^ paper contains four circular outlines printed directly on the paper as a guide to show where the biological sample should be added to the paper. For swab samples, the swab head was pressed directly onto the FTA^®^ paper using three side-to-side motions to deposit the sample. Whole blood and semen samples were obtained from healthy volunteers. For liquid samples, a total of 40 µL was pipetted onto the printed circle area of the FTA^®^ paper. During the analysis of semen samples, the use of 1 M dithiothreitol (DTT) (Sigma-Aldrich) was investigated as a reducing agent to increase sperm cell lysis. All samples were allowed to air dry for a minimum of 3 h before being placed in a desiccated storage environment until required.

### 2.3. DNA Purification Procedure

The proprietary chemicals, including a chaotropic salt, impregnated into FTA^®^ paper have inhibitory effects on the DNA amplification processes and so require removal prior to downstream analysis [13].

#### 2.3.1. Conventional Method

A 2-mm disc of FTA^®^ paper was removed from the prepared samples using a Micro-Punch (Harris) and added to a tube containing 500 µL of sterile water. Samples were then pulse vortexed 3 times for a total of 5 s before the disc was removed and ready for direct amplification.

#### 2.3.2. Microfluidic Method

The etched channels and chambers were filled with Tris-EDTA (TE) buffer (10 mM Tris (Sigma-Aldrich), 0.1 mM ethylenediaminetetraacetic acid (EDTA) (Sigma-Aldrich), adjusted to pH 8.0). A 2-mm disc of FTA^®^ paper was removed from the prepared samples and placed within the central chamber of the microfluidic device. Platinum electrodes, connected to an external Paragon 3B Power Supply Unit (Kingfield Electronics, Chesterfield, UK), were then placed into the reagent and waste wells. Purification was achieved through EOF of the TE buffer over the FTA^®^ paper, using an applied voltage of between 50 and 150 Vcm^−1^.

### 2.4. DNA Quantification

DNA quantification was performed on a Multiskan™ GO Microplate Spectrophotometer (Thermo Scientific, Cramlington, UK) according to the manufacturer’s instructions.

### 2.5. DNA Amplification Procedure

DNA amplification was carried out using the following PCR reagents: 1× GoTaq^®^ buffer, 2 mM MgCl_2_, 1 unit GoTaq^®^ HotStart DNA polymerase (Promega, Southampton, UK), 10 mg·mL^−1^ bovine serum albumin (NEB Inc., Hitchin, UK), 0.01% (*w*/*v*) poly(vinylpyrrolidone), 0.1% (*v*/*v*) Tween-20 (Sigma-Aldrich), 200 µM each deoxyribonucleotide triphosphates (Bioline, London, UK) and 0.1 µM D21 S11 forward (5′-TGTATTAGTCAATGTTCTCCAGAGAC-3′) and reverse primers (5′-ATATGTGAGTCAATTCCC-CAAG-3′) (Eurofins MWG Operon, Regensburg, Germany) [25]. D21 S11 is an example short tandem repeat locus used in a range of forensic DNA profiling kits, such as ESI 17 (Promega). It has a wide allele range (12–41.2) and therefore is a powerful tool for discrimination between individuals [26]. For real-time quantitative PCR (qPCR), PCR products were detected using SensiFAST™ SYBR Lo-ROX Mix (Bioline), an alternative HotStart DNA polymerase containing SYBR^®^ Green I as the DNA intercalating dye and ROX as an optional passive reference standard.

In order to facilitate PCR reagent storage on the microfluidic device, 1.5 µL of a 10× concentrated PCR reagent solution was added to the recess in the central chamber, enabling the correct working concentrations to be achieved upon release and mixing with the TE buffer. The PCR reagents were then covered in a thin layer, approximately 150 µm, of low melting temperature (35–37 °C) eicosane wax (Sigma-Aldrich). The channels and chambers of the microfluidic device were then filled with liquid reagents and the FTA^®^ disc (see the DNA purification procedure and Figure 1c).

Thermal cycling was performed using a thermoelectric Peltier element, which provided both the heating and cooling required. The following program was used: initial denaturation at 94 °C for 5 min, 35 cycles of 94 °C for 30 s, 60 °C for 30 s and 72 °C for 30 s, with a final extension step of 60 °C for 7 min. Control PCR samples were also run on a Q-cycler 96 thermal cycler (Hain Lifesciences Ltd., Byfleet, UK) or a Mx3000P qPCR system (Agilent Technologies, Edinburgh, UK) for DNA quantification using the same thermal cycling parameters. All amplified DNA samples were analysed off-chip using standard gel electrophoresis techniques.

## 3. Results and Discussion

### 3.1. Optimisation of Electro-Osmotic Movement

**Figure 2:**
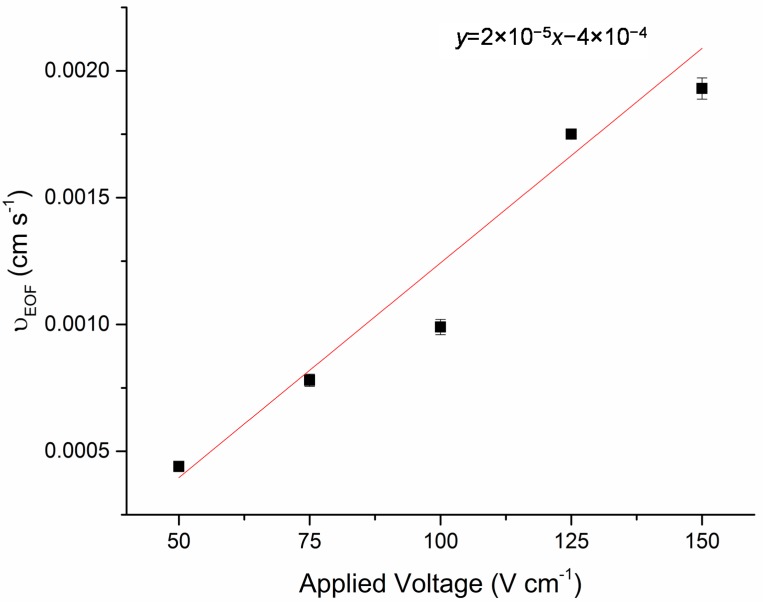
Graph showing the EOF velocity of the TE buffer used in the DNA purification process. Error bars represent the standard deviation of the triplicate analysis performed on three separate microfluidic devices.

The feasibility of manipulating the necessary reagents for DNA purification using EOF was demonstrated. The average mobility due to EOF for TE buffer was 1.1 × 10^−5^cm^2^V^−1^s^−1^ (Figure 2).

**Figure 3:**
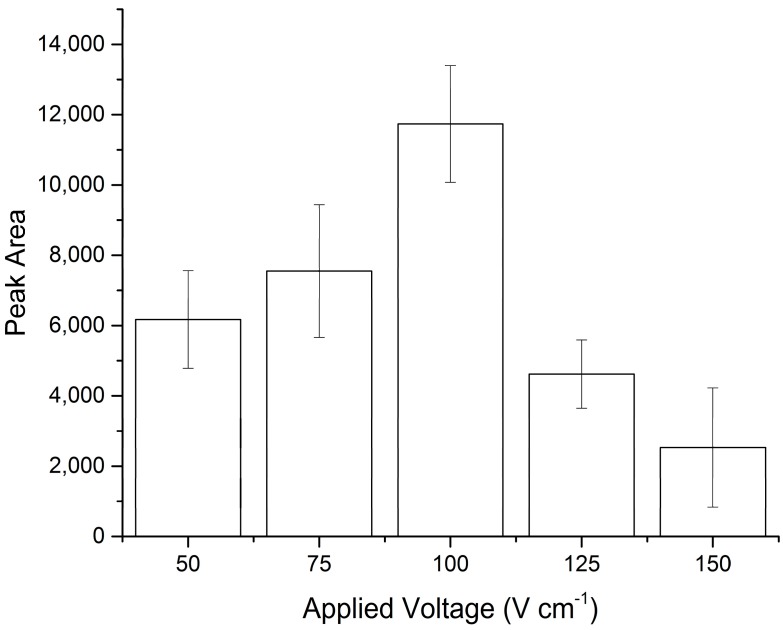
PCR product intensity compared to the voltage applied during the purification procedure on the microfluidic device (*n* = 3). Peak area refers to the band intensity/peak area of the PCR products on the gel. Conventional off-chip positive and negative controls produced peak areas of 4768 (±780) and 93 (±93), respectively. Error bars represent the standard deviation from triplicate analysis.

Optimisation of the applied voltage was necessary to ensure that potential PCR inhibitors were successfully removed from the FTA^®^ paper, but that the DNA remained in place. Voltages ranging from 50 to 150 Vcm^−1^ were tested (Figure 3), with 100 Vcm^−1^ producing significantly better results in terms of PCR product intensity following amplification, indicative of a greater quantity/quality of the starting template (*p* < 0.001, one-way analysis of variance (ANOVA) and confirmed using Tukey’s post hoc test, 95% confidence interval).

In order to establish the efficiency of the microfluidic process, quantification using qPCR was performed on FTA^®^ paper discs, which had been spiked with 25 ng of pre-purified DNA (buccal cells extracted using a QIAamp DNA Micro Kit (Qiagen, Manchester, UK), quantified using a Multiskan™ GO Microplate Spectrophotometer (Thermo Scientific)) and subjected to different voltages. In addition, samples were also collected from the anode and cathode wells to check for movement of DNA to evaluate if the use of EOF, at different applied voltages, results in any loss of DNA from the FTA^®^ paper (Table 1). The results confirm the initial results, which show 100 Vcm^−1^ to be optimum. It is hypothesised that at the lower voltages, DNA amplification efficiency is reduced due to the remaining presence of some inhibitors, while at the higher voltages, DNA is being lost from the FTA^®^ paper due to the strong attraction to the anode.

### 3.2. Integrated DNA Purification and Amplification

Successful encapsulation of concentrated PCR reagents enabled both DNA purification and amplification to be performed in a single chamber on the microfluidic device. Concentrated PCR reagents were located in the recess of the central chamber encapsulated under a layer of eicosane (optimum 30% *v*/*v*; see Electronic Supplementary Information (ESI) Figure S1 for more details). The FTA^®^ disc was placed on top of the wax layer, in plane with the microfluidic channels, allowing washing of the FTA^®^ disc with TE buffer. Following DNA purification using the optimized parameters, PCR reagents were released during the initial denaturing step due to melting of the eicosane layer, leading to the dissolution of the DNA amplification reagents in the TE buffer. PCR products were analysed off-chip by capillary gel electrophoresis, which confirmed that successful DNA amplification had taken place, showing PCR products at 223 and 227 bp, within the expected range of alleles for the D21 S11 locus (see Figure S2, ESI).

### 3.3. Analysis of Different Sample Types

**Figure 4:**
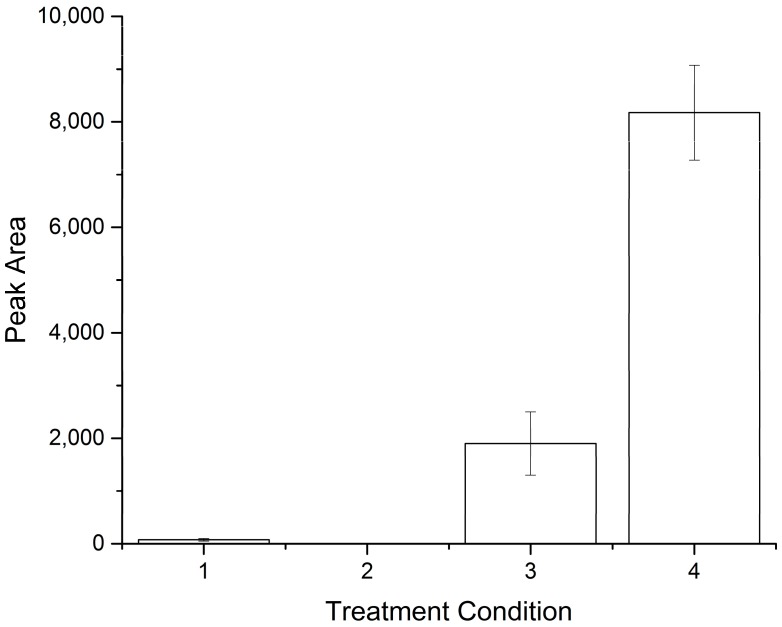
Results from direct analysis of semen samples on FTA^®^ paper using a variety of treatment conditions for disulphide bond reduction (*n* = 3). A range of treatment options were tested: (1) semen added to FTA^®^ paper and dried; (2) semen added to FTA^®^ paper, dried, 40 µL of 1 M DTT added and dried; (3) semen and 1 M DTT mixed 50:50 (*v*/*v*), added to FTA^®^ paper and dried; (4) 40 µL of 1 M DTT added to FTA^®^ paper, dried, semen added and dried. Error bars represent the standard deviation from triplicate analysis.

Buccal swabs, semen and blood samples represent some of the most commonly-encountered biological matrices in clinical or forensic settings and have different properties that any purification system needs to be able to handle prior to genetic analysis. DNA amplification was readily achieved from buccal swabs and whole blood samples, but not from semen samples (see ESI, Figures S3 and S4). In order to achieve maximum results from the semen samples, additional treatment with dithiothreitol (DTT) is required to reduce the large number of disulphide bonds present in the sperm cell membrane [27]. A range of treatment options were tested, and the order in which DTT was added was found to affect how efficient sperm cell lysis was. Pre-treating the FTA^®^ paper with 1 M DTT prior to sample addition facilitated the release of significantly more DNA from the semen sample (*p* < 0.001, one-way ANOVA confirmed using Tukey’s post hoc test, 95% confidence interval), as well as being the simplest to implement operationally (Figure 4 and Figure S5 (ESI)).

## 4. Conclusions

The work presented here demonstrates the successful integration of DNA purification and amplification processes on a single microfluidic device. Direct inclusion of FTA^®^ discs within the system provides a simple real-world interface that exploits the Micro-Punch to facilitate the addition of the biological samples into the microfluidic device. The system has been shown to provide a flexible interface for the analysis of buccal, whole blood and semen samples. The inherent advantages of using FTA^®^ paper also make the proposed system ideal for the analysis of archived biological samples. Using EOF in place of more traditional hydrodynamic pumping mechanisms eliminates any moving parts from the reagent transport mechanism and, thus, simplifies the complexity of the design and footprint of the overall microfluidic system. By combining both DNA purification and amplification techniques on a single device, the risk of sample contamination would most likely be reduced.

## Figures and Tables

**Table 1:** Results showing the amounts of qPCR products formed as a percentage of the original template DNA added, which are amplified from different locations (FTA^®^ paper disc, anode and cathode reservoirs) when FTA^®^ discs were subjected to a range of voltages for DNA purification on microfluidic devices.

Applied Voltage (Vcm^−1^)	50	75	100	125	150
FTA^®^ paper	77.8%	83.8%	87.3%	42.9%	29.8%
Anode	-	-	-	17.3%	26.8%
Cathode	-	-	-	-	-

“-“ Indicates no DNA was detected. Negative controls were also performed and produced a null (-) result.

## Supplementary Materials

The following are available online at http://www.mdpi.com/2072-666X/7/7/119/s1, Figure S1: Graph showing the effect of the inclusion of wax within the PCR reagent solution. Samples were subjected to conventional DNA amplification and analysed by gel electrophoresis (*n* = 3). Error bars represent the standard deviation from triplicate analysis, Figure S2: Electropherogram showing PCR products from the amplification of the D21 S11 locus, using the microfluidic system for integrated DNA purification and amplification, as confirmed on an ABi3500 Genetic Analyser (Applied Biosystems, Thermo Fisher Scientific, Foster City, CA, USA) using a GeneScanTM 500 LIZ® Size Standard (*n* = 3), Figure S3: Graph comparing the relative efficiency of DNA amplification from a range of biological sample types (buccal swabs, semen samples and whole blood) analysed on the microfluidic device (*n* ≥ 3). Error bars represent the standard deviation of, at minimum, triplicate analysis, Figure S4: Example gel electrophoresis image showing DNA amplification from a range of biological sample types: Lane 1, DNA size ladder; 2/3, whole blood; 4/5, semen samples; 6/7, buccal swabs; 8, negative control, Figure S5: Example gel electrophoresis image showing DNA amplification from semen samples subject to a range of different dithiothreitol (DTT) treatments on FTA® paper: Lane 1, DNA size ladder; 2/3, 40 μL of 1 M DTT added to FTA® paper, dried, semen added and dried; 4/5, semen added to FTA® paper, dried, 40 μL of 1 M DTT added and dried; 6/7, semen and 1 M DTT mixed 50:50 (*v*/*v*), added to FTA® paper and dried; 8, negative control.

## Abbreviations

The following abbreviations are used in this manuscript:

DNA	Deoxyribonucleic acid
DTT	Dithiothreitol
EDTA	Ethylenediaminetetraacetic acid
EOF	Electro-osmotic flow
PCR	Polymerase chain reaction
qPCR	Real-time quantitative PCR
TE	Tris/EDTA buffer
