# Supplementary material for: Combining Electro-Osmotic Flow and FTA® Paper for DNA Analysis on Microfluidic Devices
Source: Micromachines (Basel). 2016 Jul 14;7(7):119. doi: 10.3390/mi7070119 (PMC6190317; doi:10.3390/mi7070119)
Supplement: Supplementary file 1 [file micromachines-07-00119-s001.pdf]

Supplementary Materials: Combining Electro-Osmotic Flow and FTA® Paper for DNA Analysis on Microfluidic Devices

Ryan Wimbles, Louise M. Melling and Kirsty J. Shaw

1. Optimisation of PCR Reagent Encapsulation

The effects of wax encapsulation of the polymerase chain reaction (PCR) reagents were evaluated by combining varying amounts of eicosane (0%–40% (*v*/*v*)) with the standard PCR reagent mixture. The optimum amount of wax was found to be 30% of the total reaction volume; here, the protective effect of the wax was optimal without any deleterious effects on PCR efficiency (Figure S1).

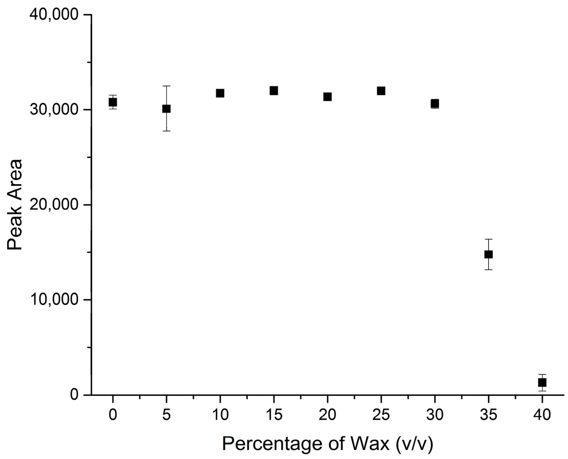

Figure S1. Graph showing the effect of the inclusion of wax within the PCR reagent solution. Samples were subjected to conventional DNA amplification and analysed by gel electrophoresis ($n = 3$). Error bars represent the standard deviation from triplicate analysis.

2. Integrated DNA Purification and Amplification

PCR products were analysed off-chip by capillary gel electrophoresis, confirming successful DNA amplification showing PCR products at 223 and 227 bp (Figure S2).

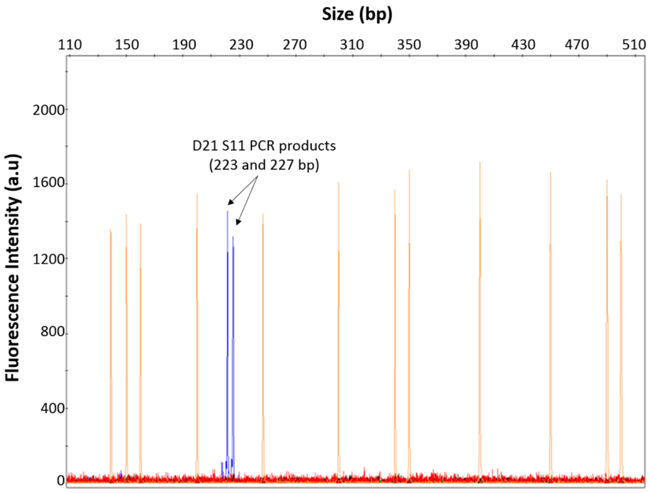

Figure S2. Electropherogram showing PCR products from the amplification of the D21 S11 locus, using the microfluidic system for integrated DNA purification and amplification, as confirmed on an ABi3500 Genetic Analyser (Applied Biosystems, Thermo Fisher Scientific, Foster City, CA, USA) using a GeneScan™ 500 LIZ® Size Standard ($n = 3$).

3. Analysis of Different Sample Types

DNA amplification was readily achieved from buccal swabs and whole blood samples, but not from semen samples, producing PCR products of the expected size range (Figure S3). Example gel electrophoresis images are included for a range of biological sample types (Figure S4) and semen treatment methods (Figure S5).

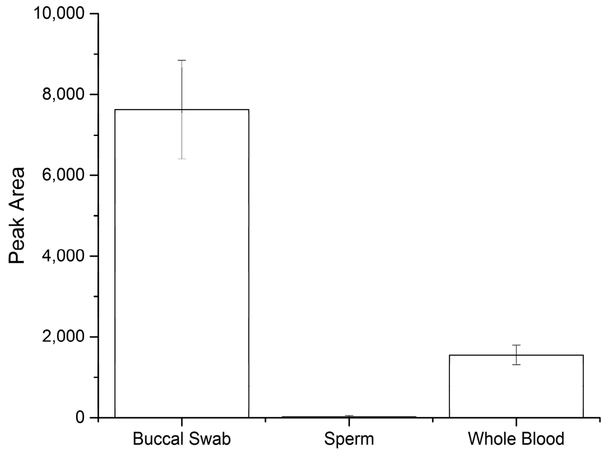

Figure S3. Graph comparing the relative efficiency of DNA amplification from a range of biological sample types (buccal swabs, semen samples and whole blood) analysed on the microfluidic device ($n \geq 3$). Error bars represent the standard deviation of, at minimum, triplicate analysis.

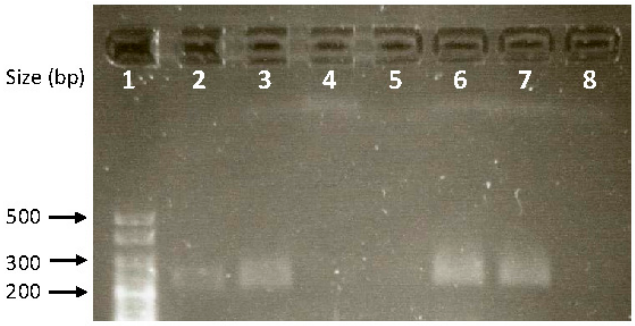

Figure S4. Example gel electrophoresis image showing DNA amplification from a range of biological sample types: Lane 1, DNA size ladder; 2/3, whole blood; 4/5, semen samples; 6/7, buccal swabs; 8, negative control.

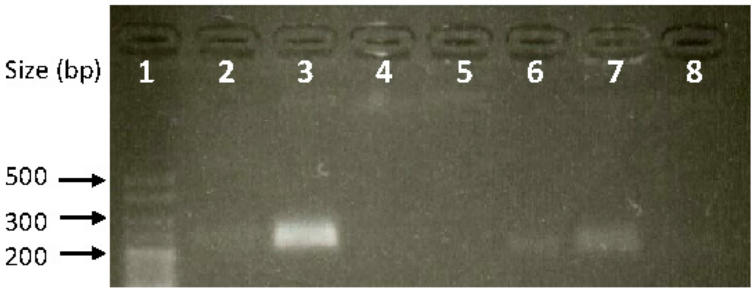

Figure S5. Example gel electrophoresis image showing DNA amplification from semen samples subject to a range of different dithiothreitol (DTT) treatments on FTA® paper: Lane 1, DNA size ladder; 2/3, 40 μL of 1 M DTT added to FTA® paper, dried, semen added and dried; 4/5, semen added to FTA® paper, dried, 40 μL of 1 M DTT added and dried; 6/7, semen and 1 M DTT mixed 50:50 (*v*/*v*), added to FTA® paper and dried; 8, negative control.
